# Supplementary material for: The Prevalence and Correlates of Suicidal Ideation, Plans and Suicide Attempts among 15- to 69-Year-Old Persons in Eswatini
Source: Behav Sci (Basel). 2020 Nov 10;10(11):172. doi: 10.3390/bs10110172 (PMC7696382; doi:10.3390/bs10110172)
Supplement: Supplementary file 1 [file behavsci-10-00172-s001.pdf]

Supplementary file Table S1: Variable information

Variables	Question	Response options (coding scheme)
	**Outcome variables**	
Suicidal ideation (past 12 months)	"During the **past 12 months**, have you seriously **considered** attempting suicide?"	"Yes, No"
Help seeking	"Did you seek **professional help** for these thoughts?"	"Yes, No"
Suicide plan (past 12 months)	"During the **past 12 months**, have you made **a plan about how** you would attempt suicide?"	"Yes, No"
Ever suicide attempt	"Have you **ever attempted suicide**?"	"Yes, No"
Suicide attempt (past 12 months)	"During the **past 12 months**, have you **attempted suicide**?"	"Yes, No"
Suicide method	"What was the main **method you used** the last time you attempted suicide?"	For example, "poisoning with pesticites (e.g., rat poison, insecticide, weed-killer)"
Medical care	"Did you seek **medical care** for this attempt?"	"Yes, No"
	"Were you **admitted to hospital overnight** because of this attempt?"	"Yes, No"
	**Psychosocial distress**	
Childhood physical abuse	"Looking back on your childhood (before age 18 years), did a parent or adult in the household ever push, grab, shove, slap, hit, burn, or throw something at you?"	1=never to 5=almost daily (coded 1=0 and 2-5=1)
Childhood sexual abuse	"Looking back on your childhood, did an adult or anyone at least five years older than you ever touch you sexually or try to make you touch them sexually or force you to have sex?"	"Yes, No"
Adult sexual abuse	"Since your 18th birthday, have you ever experienced a sex act involving either vaginal, oral, or anal penetration **against your will**?"	1=never to 4=many times (4 or more times) (coded 1=0 and 2-4=1)
Threats	"In the past 12 months, have you been frightened for the safety of yourself or your family because of the anger or threats of another person(s)?"	"Yes, No"
Alcohol family problem	"During the **past 12 months**, have you had family problems or problems with your partner due to **someone else's** drinking?"	1=yes, more than monthly to 4=yes, once or twice and 5=no (1-4=1 and 5=0)
Violent injury	"In the past 12 months, how many times were you in a violent incident in which you were injured and required medical attention?"	1=never to 4=often (6 or more times) (coded 1=0 and 2-4=1)
Family member attempted suicide	"Has anyone in **your close family** (mother, father, brother, sister or children) ever attempted suicide?"	"Yes, No"
Family member died from suicide	"Has anyone in **your close family** (mother, father, brother, sister or children) ever died from suicide?"	"Yes, No"
